# Is a community still a community? Reviewing definitions of key terms in community ecology

**DOI:** 10.1002/ece3.1651

**Published:** 2015-10-07

**Authors:** James T. Stroud, Michael R. Bush, Mark C. Ladd, Robert J. Nowicki, Andrew A. Shantz, Jennifer Sweatman

**Affiliations:** ^1^ Department of Biological SciencesFlorida International UniversityMiamiFloridaUSA; ^2^ Center for Tropical Plant ConservationFairchild Tropical Botanic GardenCoral GablesFloridaUSA

**Keywords:** Assemblage, community, community ecology, definitions, ensemble, guild

## Abstract

Community ecology is an inherently complicated field, confounded by the conflicting use of fundamental terms. Nearly two decades ago, Fauth et al. (1996) demonstrated that imprecise language led to the virtual synonymy of important terms and so attempted to clearly define four keywords in community ecology; “*community,*” “*assemblage,*” “*guild,*” and “*ensemble*”. We revisit Fauth et al.'s conclusion and discuss how the use of these terms has changed over time since their review. An updated analysis of term definition from a selection of popular ecological textbooks suggests that definitions have drifted away from those encountered pre‐1996, and slightly disagreed with results from a survey of 100 ecology professionals (comprising of academic professors, nonacademic PhDs, graduate and undergraduate biology students). Results suggest that confusion about these terms is still widespread in ecology. We conclude with clear suggestions for definitions of each term to be adopted hereafter to provide greater cohesion among research groups.

## Introduction

Ecology is a young but rapidly developing field of science. Unlike more established fields, such as mathematics and physics, ecologists are yet to create an established and unambiguous framework of terminology (Hodges 2008). Nearly two decades ago, Fauth et al. (1996) (hereafter Fauth et al.) attempted to clarify terminology in the field of community ecology, a subdiscipline of ecology that is frequently criticized for being jargon‐filled and prone to synonymy (Peters 1976; Thorp 1986; Mills et al. 1993; Frazier 1994; Morin 2011). Improper and irregular use of distinct terms generates confusion, particularly among students, that may negatively impact scientific understanding and development. It is therefore important to clearly define key terms to facilitate scientific communication, increase precision of foundational concepts and ideas, and aid the directional development of future research. A recent request for the establishment of a Convention of Ecology Nomenclature (CEN) (Herrando‐Pérez et al. 2014) highlights the widespread problem of imprecise terminology in ecology and provides well‐timed support for the utility of this review.

Despite being foundational concepts, key ecological terms such as *community* or *assemblage* are prone to subjective interpretations by ecologists. Variability in the use of these terms can impact the efficacy of comparisons across ecological datasets, which in a discipline that includes increasingly larger temporal and spatial scales may hinder the interpretation of more comprehensive ecosystem patterns (Drake 1990). Although attempts had been made to address the terminological issues in community ecology prior to Fauth et al., the introduction of new terms generally has not proven successful (e.g., *similial community*; Schoener 1986). Similarly, the problem of terminological inconsistencies in community ecology has continued to be acknowledged since Fauth et al., but rarely confronted (Wilson 1999; Morin 2011; Mittelbach 2012).

**Figure 1:**
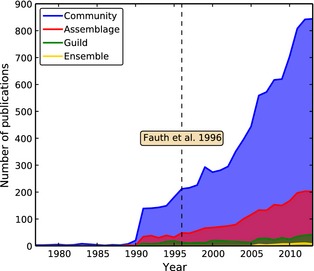
Relative interest in community ecology terms from 1977 to 2013, as reflected by respective citation histories (trends are overlayed, not stacked). The publication date of Fauth et al. is indicated by a vertical dashed line. Terms were searched for in the “ecology” category of *ISI Web of Science* (accessed 20 February 14).

Fauth et al. identified four terms of importance in the field which were prone to cause confusion, synonymy, or misuse: *community*,* assemblage*,* guild*, and *ensemble*. Over the last half a century, the field of community ecology has experienced a substantial rise in popularity, with all definitions experiencing an increased use in ecological literature since Fauth et al.'s review (Fig. 1). The popularity of the two most important terms in the field, and therefore perhaps the greatest proxy for the field's own popularity, *community* and *assemblage*, have experienced dramatic increases in use yet remain frequently misused and synonymized.

We review how these terms have been applied and defined in successive literature since 1996. Specifically, we attempt to identify long‐term trends in usage and meaning of these four terms, determine whether the interpretations of each definition have changed and evaluate the current state of variable definitions in today's ecological discipline. At the beginning of each individual terminological review, we provide our interpretation of the most broadly accepted current definition. We suggest these definitions as references for future studies of ecology.

## Present‐Day Interpretations

To estimate how these four terms are used by the contemporary ecological community, we developed a survey that asked ecologists to define the four key terms (*community*,* assemblage*,* guild*, and *ensemble*) (see Online Appendix A1 for survey questionnaire). We advertised the survey on several popular ecology blogs, mailing lists (e.g., ECOLOG list server) and social media outlets (Facebook, Twitter). During the sampling period of 7 days, approximately 400–500 people viewed the survey and provided 100 completed surveys (20–25% completion rate). We received responses from 11 countries and 31 states within the United States. Respondents were asked to report their profession (32 academic professors, 36 graduate students, 15 nonacademic PhDs, 5 undergraduate students, and 11 other) and field of study. Definitions were quantified using weighted rubrics of key words, which were designed to encompass the most important descriptive factors of each term (see Online Appendix A2). This survey was conducted in an attempt to observe if there existed differences in term understanding between students of ecology ranging from undergraduate level to professors. Additionally, we aimed to assess whether their interpretations correctly aligned with definition trends displayed in popular ecological textbooks (Table 2).

*Community*: A group of interacting species populations occurring together in space.

Ostensibly the flagship term in community ecology, *community*, is arguably the most prone to varying interpretations among ecologists (Morin 2011). Indeed, the underlying concept of what a community is and how it is organized has consistently changed through time (Roughgarden and Diamond 1986; Schoener 1986; Fauth et al. 1996; Morin 2011). In its simplest form, a community describes “all of the organisms in a prescribed area” (Roughgarden and Diamond 1986). This simple description, however, does not consider four separate features which may be important when studying ecological communities: space, time, taxa, and trophic characteristics.

The two most integral components to the structure of a community are space and time. By definition, community members must be together in space; these members must also be present in the same space at the same time for interaction – another fundamental property of communities – to occur. Properties of communities that are not defined but often used as a part of the community definition include taxonomic features, trophic characteristics, and life form associates. Often a broad higher‐level taxon outlines the central unifying theme of the community, such as when one discusses a *bird* community. A community may also be classified when a focal group of species inhabit a similar trophic position, such as plant, parasite, or carrion community (Roughgarden and Diamond 1986). We will revisit the importance of taxonomic and trophic relatedness further when discussing the terms *assemblage*,* guild,* and *functional group*.

Fauth et al. define *community* as “a set of species occurring in the same place at the same time”. We continued Fauth et al.'s review of terminological definitions of *community* in ecology textbooks post‐1996 to explore subsequent trends in author definitions (Table 2). An observed shift toward definitions which do not require species interactions is evident. This disagrees with our survey results which suggest over half of ecologists (51.58%; Table 1) consider interspecific interactions to be a key component of a *community*.

**Table 1:** Percentage of definitions falling within each rubric. Percentages for each occupation (*i.e.,* graduate students) are relative to the total number responding for that occupation alone (*i.e.,* 79.41% of graduate students (29/36) defined community with an explicit spatiotemporal component). Bold values indicate cumulative scores for each key definition

	Spatial/Temporal (%)	Taxonomic/Phylogenetic (%)	Interactions (%)	Functional Similarity (%)	Share Resources (%)	Different Species (%)	All Species (%)	Never Heard (%)
Community	**82.11**	**4.21**	**51.58**	**1.05**	**0**	**67.37**	**36.84**	**0**
Professor	90.63	3.13	50.00	0	0	75.00	31.25	0
Government/Nonprofit	84.62	0	46.15	0	0	53.85	30.77	0
Graduate Student	79.41	5.88	50.00	2.94	0	61.79	41.18	0
Undergraduate	100.00	20.00	60.00	0	0	80.00	20.00	0
Other	54.55	0	63.64	0	0	72.73	54.55	0
Assemblage	**72.63**	**33.68**	**14.74**	**8.42**	**2.11**	**81.05**	**5.26**	**2.11**
Professor	78.13	43.75	15.63	0	0	84.38	3.13	3.13
Government/Nonprofit	69.23	15.38	7.69	7.69	0	69.23	7.69	7.69
Graduate Student	70.59	41.18	20.59	20.59	0	85.29	8.82	0
Undergraduate	60.00	0	0	0	20.00	60.00	0	0
Other	72.73	18.18	9.09	0	9.09	81.82	0	0
Guild	**9.47**	**4.21**	**4.21**	**58.95**	**38.95**	**72.63**	**0**	**5.26**
Professor	6.25	3.13	0	62.50	43.75	68.75	0	3.13
Government/Nonprofit	23.08	7.69	7.69	61.54	30.77	61.54	0	7.69
Graduate Student	11.76	2.94	8.82	67.65	35.29	76.47	0	0
Undergraduate	0	20.00	0	20.00	20.00	40.00	0	60.00
Other	0	0	0	36.36	54.55	100.00	0	0
Ensemble	**21.05**	**12.63**	**4.21**	**5.26**	**5.26**	**23.16**	**0**	**35.79**
Professor	21.88	12.50	3.13	6.25	6.25	21.88	0	43.75
Government/Nonprofit	15.38	0	7.69	0	7.69	15.38	0	23.08
Graduate Student	23.53	17.65	5.88	8.82	2.94	29.41	0	29.41
Undergraduate	20.00	0	0	0	20	40.00	0	20.00
Other	18.18	18.18	0	0	0	9	0	54.55

There was acceptance among all survey groups that *community* should include a spatiotemporal aspect (82.11%; Table 1). Although many textbooks agreed that space was explicit, there was some variability when incorporating time, indicating a shift in definition since 1996 (Table 2). Survey data revealed that respondents believed that a *community* should include multiple different species (67.37%; Table 1); however, agreement that a community should contain all species in a given area received less support (36.84%; Table 1). There was weak support for a phylogenetic component of the definition (4.21%; Table 1), which was supported by definitions in ecological textbooks (Table 2). Graduate students and professors displayed weak support for a phylogenetic or taxonomic basis for the definition of *community* (5.88% and 3.13%, respectively), compared to 20% of undergraduates surveyed. This suggests an understanding of basic ecology terminology that is adjusted with career progression, but also provides the potential for a misunderstanding of basic definitions that may persevere over the span of a career.

**Table 2:** Comparison of definitions of *community* taken directly from glossary (or if stated definitively in text) of key ecology textbooks. Data are included from Fauth et al. (1996) and a subsequent review of ecological textbooks post‐1996

Set boundaries	Definition	Source
Pre‐1996 (from Fauth et al.)		
Space, time	The species that occur together in space and time	Begon et al. (1990)
Space, time, interactions	An association of interacting populations, usually defined by the nature of their interaction or the place in which they live	Ricklefs (1990)
	A group of organisms that live alongside one another, and in which the different species and individuals interact with one another	Tudge (1991)
Space, time, interactions, phylogeny	A group of interacting plants and animals inhabiting a given area	Smith (1992)
	An assemblage of interacting plants and animals on a shared site	Freedman (1989)
	Group of populations of plants and animals in a given place; ecological unit used in a broad sense to include groups of various sizes and degrees of integration	Krebs (1985)
Post‐1996		
Space	The collection of species found in a particular place	Morin (2011)
Space, phylogeny	The total living biotic component of an ecosystem, including plants, animals and microbes.	Calow (2009)
	A group of populations of plants and animals in a given place; used in a broad sense to refer to ecological units of various sizes and degrees of integration	Stiling (1996)
Space, interaction	A group of species living together and interacting through ecological processes such as competition and predation	Levinton (2009)
	An association of interacting populations, usually defined by the nature of their interaction or by the place in which they live	Ricklefs and Miller (1999)
	An association of interacting species living in a particular area; also often defined as all of the organisms living in a particular area	Molles (2010)
Space, time	The species that occur together in space and time	Begon et al. (21990)
	All the species of organisms found in a defined area over ecological time	Dodds (2009)
Space, time, interactions	An assemblage of interacting populations forming and identifiable group within a biome	Arora and Kanta (2009)
Space, time, interactions, phylogeny	n/a	

We argue that the commonly accepted use of *community* as a “group of species that occur together in space and time” (Begon et al. 1990; Mittelbach 2012), although effective, is too broad and hard to distinguish from assemblage.

We agree with Schoener (1986) that broadness will aid in the simplicity and flexibility of the use of the term community. However, to break synonymy with assemblage, we amend Begon et al.'s (2006) definition to “a group of interacting species populations occurring together in space”, for example, a *lowland forest community*. Although species interactions are often considered nonessential to the definition of a community, but rather provide a hypothesis to be tested, we argue that interactions, both direct and indirect, are a fundamental component of a community. Direct interactions between species can lead to important indirect consequences. For example, the trophic cascade hypothesis has been the subject of increased study over the last several decades; direct predator–prey interactions can result indirectly in population increases or decreases of other species in the community (Pace et al. 1999). Acknowledging these interactions, both direct and indirect, also provide a further axis on which to discriminate between community and assemblage or guild, which do not explicitly require them. Similarly, having an explicit temporal constraint on species’ existence in a community may be confused by immigration and emigration dynamics. This may result in the separation of a large dynamic community into multiple smaller stable communities, a point particularly valid when considered within the context of metacommunity ecology.

### The Rise of Metacommunity Ecology

Spatial and temporal scaling is one of the most challenging aspects of ecology. Within the field of community ecology, many focal study communities are nested within a larger community. Therefore, the bounds of the study community are usually artificial and defined by the researcher. Historically, this resulted in the study of communities at a small spatial scale to allow for comprehensive assessments of observed patterns. However, recent interest in studying interactions between communities and how these may affect the underlying dynamics of multiple spatially explicit communities has led to the rise of metacommunity ecology.

Perhaps no recent development in community ecology has been as great as the establishment of the term “metacommunity”, defined as a group of interacting communities that are connected through the dispersal of multiple species (Wilson 1992). This term formally recognizes the role that scale, both spatial and temporal, has on the function of community dynamics. While the definition of “metapopulation”, or a set of dispersal‐linked populations (Gilpin and Hanski 1991), implicitly incorporated scale into its theoretical framework, the complexity of research in community ecology assumes varying dispersal rates and levels of connectivity among species within the defined community. To simplify the study of communities into manageable experiments, communities were previously viewed as a closed group of interacting species, isolated from other communities. While the metacommunity framework recognizes that dispersal might be important in community interactions, it has also led to the establishment of a series of operating paradigms that might be affecting the functional dynamics of a set of communities. These paradigms, for example, patch‐dynamics, species‐sorting, mass‐effects, and neutral theory, all involve variation in either species’ behavior, space or time that allows for the persistence of interlinked communities (see Leibold et al. 2004 for detailed review).

Hubbell's neutral model (Hubbell 2001), a widely used null model for examining community structure, has received considerable interest in community ecology and spurred the growth of lively ecological debate and empirical examination in a relatively short amount of time (McGill 2003; Harpole and Tilman 2006; Alonso et al. 2006). Perhaps the most powerful feature of the metacommunity framework is the recognition that these paradigms are not mutually exclusive, but that they may exist in a gradient that can also vary with temporal scale. This allows for seasonal processes such as seed dispersal or larvae production to be incorporated into community studies which may have previously been ignored if rigid temporal constraints on the definition of a community were employed.

*Assemblage*: A taxonomically related group of species populations that occur together in space.

In recent ecology textbooks the term appears frequently though is rarely defined (e.g., Ricklefs 2007; Molles 2009), prompting some confusion about its distinctiveness from *community*. Specifically, a common difference is the structural framework within which an assemblage is set – often either as a subset of a community (Fauth et al. 1996) or as an independent geographic area (Ricklefs and Miller 1999). While similar to the definition provided by Fauth et al., by replacing the term *community* with “geographic area,” Ricklefs and Miller (1999) avoid confusion that may stem from multiple definitions of *community* (Ricklefs and Miller 1999; Carson and Schnitzer 2008; Molles 2009; Morin 2011). There was strong support for the low importance of species interactions in the definition of assemblage, only 14.74% of respondents considered it important (Table 1).

There was a clear distinction in interpretation by ecologists between *community* and *assemblage* (Table 1), with evidence that taxonomic or phylogenetic information is much more heavily associated with *assemblage* (33.68%; Table 1) than with *community* (4.21%; Table 1). Most surveyors also recognized that *assemblage* should refer to a group of different species (81.05%; Table 1), but not necessarily all species (5.26%; Table 1), in a geographic area. Additionally, a high proportion of those surveyed (72.63%; Table 1) identified *assemblage* as having a spatial or temporal component. There was confusion between whether species in an *assemblage* share resources, with moderate support by undergraduate students (20%; Table 1) in comparison with either professors or graduate students (0%, respectively; Table 1).

We suggest that *assemblage* should act as a taxonomically restricted correlate of the term *community*, for example, a *lowland forest amphibian assemblage*. We propose that the clearest and most comprehensive definition of *assemblage* should be “a taxonomically related group of species that occur together in space and time.” The highest taxonomic grouping to which this term should be applied is Class, for example, *Aves* if discussing a bird community. Interactions within assemblages can occur; however, we think that they are not explicitly required and therefore are not included in the definition. Clarification in the definitions of *community* and *assemblage* terms will benefit ecology as a whole by allowing for an increased potential in cross‐literature comparisons and future meta‐analyses.

### On the Use of Community Terms in Macroecology

Certain subfields of ecology, such as ecosystems and community ecology, seek to understand how abiotic (e.g., climatic) and biological (e.g., phylogenetic) processes drive ecological processes and patterns (Keith et al. 2012). This has led to the development of a new subfield; macroecology, which aims to assess how both biotic and abiotic characteristics influence patterns of species diversity across different spatial scales. For example, studies of biodiversity–ecosystem functioning (BEF) aim to assess how biodiversity influences ecosystem characteristics such as biomass production or carbon sequestration. While a multitude of evidence has shown a positive relationship between biodiversity and ecosystem function (see Cardinale et al. 2012 for a review), the contribution of individual species can vary substantially and subsequently be difficult to predict (Walker 1992; Walker 1995; Peterson^1998^). Attempting to identify functional roles of species, or groups of species, in communities may provide an opportunity to better understand the relationship between species diversity and ecosystem functioning. To facilitate this, the terms *guild* and *functional group* have been employed to describe functionally similar groups of species; however, the distinction between the two is unclear which can lead to erroneous synonymy.

*Guild:* A group of species that exploit the same class of resources in a similar way.

Fauth et al. defined guilds as “a group of species without regard for taxonomic position that exploit the same class of environmental resources in a similar way”. This definition is based on that of Root (1967), with the assumption that similar resource use by species in a *guild* then makes that resource unavailable for use by others. The term *guild* has maintained a fairly consistent definition in ecology since its inception (Root 1967; Morin 2011); however, it is frequently used restrictively from a trophic perspective, such as when food is the shared resource (Stiling 1996; Arora and Kanta 2009).

Survey results indicate that the most important defining factor of a *guild* was the functional similarity (58.95%; Table 1) between the different species (72.63%; Table 1) of component species in the group. Functional similarity of component species was more commonly associated with the definition of *guild* than the sharing of resources by component species (38.95%; Table 1). There was weak support both for taxonomic/phylogenetic factors and species interactions as central features of the definition of *guild* (4.21%, respectively; Table 1). Surprisingly, some respondents had never heard of the term *guild*, which was particularly true of undergraduate ecology students (overall mean = 5.26%; professors = 3.13%; undergraduates = 60%).

We support the current generalized use of this broad term to reflect a group of species utilizing a shared resource. We suggest Calow's (2009) definition as the most clear and concise; “a group of species that exploit the same class of resources in a similar way”. This implies that phylogeny is not a fundamental aspect of the definition. We propose the split of *guild* into two subdefinitions, *functional guild* and *taxonomic guild*, so as not to repeat the patterns of generalized and ambiguous interpretations that have been observed in the use of the term *community*. We suggest *taxonomic guild* refer to “a group of *taxonomically related* species that exploit the same class of resources in a similar way”, while *functional guild* refers more broadly to “a group of *functionally similar* species that exploit the same class of resources in a similar way*”* (Gitay and Noble 1997; Fargione et al. 2003; Manzaneda and Rey 2008).

*Functional group*, however, is a broader term with variable definitions, which encompass species traits, processes, and functions (Violle et al. 2007; Krebs 2008; Levinton 2009; Morin 2011) that are generally not spatially defined (although see Krebs 2008). There are no explicit restrictions on taxonomy; however, such restrictions can occur implicitly based on the functional group of interest (e.g., Nitrogen fixers). By this definition, guilds are a specialized kind of functional group centered on resource use and its associated processes. This can be confused by the membership of certain species to more than one functional group despite being classified in the same guild. For example, predatory birds and predatory cetaceans may belong to the same guild (piscivores) within the same ecosystem, but belong to different functional groups as one subgroup subsidizes nutrients to terrestrial systems (predatory seabirds; Anderson and Polis 1999) while the other remains aquatic (predatory cetaceans; Degrati et al. 2014).

Functional groups often necessarily encompass other functional traits beyond the trait of primary interest: for example, seagrasses have impacts on their community by stabilizing sediment and baffling water currents (a geophysical process; Dennison 1993), sequestering carbon (a biogeochemical process; Fourqurean et al. 2012), providing food for herbivores (a trophic process; Valentine and Heck 1999; Heck and Valentine 2006), and generating habitat complexity (a structural process; Heck et al. 2003; Duffy 2006). Additionally, these processes can be facultative, competitive, or inhibitive. As a result, individual seagrass species may differ in the relative contributions they make to each of these community processes and can be simultaneously placed in multiple, and sometimes contrary, functional groups. Indeed, the placement of individual species into functional groups is fluid, highly dependent on the question of interest, and not necessarily correlative with taxonomy.

### Incorporating Phylogeny Into Functional Groups

One pattern that has emerged repeatedly is that phylogenetically similar species can have very different functional roles (Duffy 2006). For example, closely related parrotfish (family: *Scaridae*) on Pacific and Caribbean reefs have very different functional roles depending on whether they bioerode reefs or not (Bellwood and Choat 1990). Indeed, diversity at any level can potentially affect ecosystem processes (Duffy 2006). To some extent, individual specialization within a species may complicate the placement of even a single species into a cogent functional group (Vander Zanden et al. 2010; Matich et al. 2011). We suggest that instead of taxonomy being used as a measure of functional similarity, it should be used primarily as a tool to examine environmental limitations and physiological adaptations of a group. These abiotic or physiological factors, sometimes formally described as functional traits (Violle et al. 2007), are commonly used in both macroecology and the BEF literature. In macroecology, inclusion of taxonomy can lead to better insights into the physiological and geographic limitations of functional groups, which may aid identification of important interactions and patterns in distribution of functional groups through space and time. A focus on biotic and abiotic interactions, which a coherent concept of functional group would aid, is suggested to be an important component of understanding processes that drive patterns in macroecology (Keith et al. 2012). In the BEF literature, informing how a group will react to changes in the environment is an important component of stability mechanics (*sensu* Cardinale et al. 2012), and the stability of functional groups has implications for the stability of ecosystems as a whole (Bellwood et al. 2004). We therefore suggest the “functional” aspect of the group should be used to group species based on how they affect the environment (i.e., bioeroders), while taxonomic delineations (e.g., family *Scaridae*) should be used to inform environmental and physiological constraints.

## Ensemble

The use of *ensemble* in ecology remains rare, although the term has experienced an increase in use over the past decade (Fig. 1). *Ensemble* was of exceptional significance in our survey as many ecologists had either not heard of it being used in an ecological context (35.79%; Table 1), had never used it, or considered it synonymous with assemblage. Fauth et al. defined ensemble as “*a phylogenetically bounded group of species that use a similar set of resources within a community*”.

There was widespread confusion of the true definition of *ensemble* among surveyed ecologists, with no single defining factor gaining support from more than 25% of respondents. Additionally, although used in past literature (e.g., Istock 1973), we did not find a single inclusion of *ensemble* in the glossary of any ecological textbook included in our literature review.

We commend Fauth et al. for attempting to establish *ensemble* in community ecology to aid in the development of terminological clarity; however, we argue that *ensemble* has as rarely been properly recognized or used in a correct ecological context and, therefore, propose that it is redundant. The current widespread misunderstanding of its use and definition in modern ecology supports this assertion (Table 1, 2).

## Conclusion

This article aims to build on previous attempts of terminological standardization. We applaud earlier attempts (e.g., Fauth et al.) at conceptualizing these terms; however, feel that the current usage of all terms has continued to deviate despite these efforts, and an updated review was needed. Remarkably, there was no complete agreement for any one definitive factor for any term in our survey of ecologists (range = 0–82.11%). This highlights that, although incredibly popular in current literature (Fig. 1), there remains some variability in term interpretation.

Here, similar to Schoener (1986), we have attempted to clarify current terms rather than propose new ones as we felt reluctant to add more terms to an already jargon‐filled field. We hope this review aids in the continued growth of ecology and serves as a point of reference for definitive summaries of fundamental terms.

## Conflict of Interest

None declared.

## Supporting information


**Table S1.** Survey questionnaire. **Table S2.** Survey results quantification methods.
