# Supplementary material for: Is a community still a community? Reviewing definitions of key terms in community ecology
Source: Ecol Evol. 2015 Oct 7;5(21):4757–65. doi: 10.1002/ece3.1651 (PMC4662321; doi:10.1002/ece3.1651)
Supplement: Supplementary file 1 — Table S1. Survey questionnaire. Table S2. Survey results quantification methods. [file ECE3-5-4757-s001.docx]

**SUPPORTING INFORMATION**

**Table A1; Survey questionnaire**

| 1. | Define community |  |  |
| --- | --- | --- | --- |
| 2. | Define assemblage |  |  |
| 3. | Define guild |  |  |
| 4. | Define ensemble |  |  |
| 5. | What is your profession? |  | Professor |
|  | (please choose one) |  | Government/non-profit |
|  |  |  | Graduate student |
|  |  |  | Undergraduate |
| 6. | What is your field of study? |  |  |

**Table A2; Survey results quantification methods**

| Key words/term | Context |
| --- | --- |
|  |  |
| Spatio/Temporal | Requires a spatial or temporal component |
| Taxonomic/Phylogenetic Relatedness | Requires a taxonomic or phylogenetic component |
| Interactions | Requires interactions among species, either direct  or indirect |
| Functional Similarity | Requires species to possess similar traits or be  functionally similar |
| Share Resources | Requires that species share resources |
| Different Species | Requires multiple species |
| All Species | Definition encompassess all species in some area versus  a subset of species |
| Never Heard | The respondent had never heard the term |

All rubrics require explicit language. For example, "spatial/temporal" requires explicit declaration of a spatial or temporal component. That is "all species in a food web" is NOT spatially explicit (because food webs can be arbitrarily large, depending on how one defines the edges). For all categories 1 = TRUE, 0 = FALSE unless otherwise stated. NOTE: for "different species" 0 can include definitions that require multiple populations, but don't explicitly state multiple species.
